# Supplementary material for: Comparative Genomic Analysis of Fusobacterium necrophorum Provides Insights into Conserved Virulence Genes
Source: Microbiol Spectr. 2022 Oct 7;10(6):e00297-22. doi: 10.1128/spectrum.00297-22 (PMC9769765; doi:10.1128/spectrum.00297-22)
Supplement: Supplementary file 9 — Fig. S1. Download spectrum.00297-22-s0009.pdf, PDF file, 0.2 MB [file spectrum.00297-22-s0009.pdf]

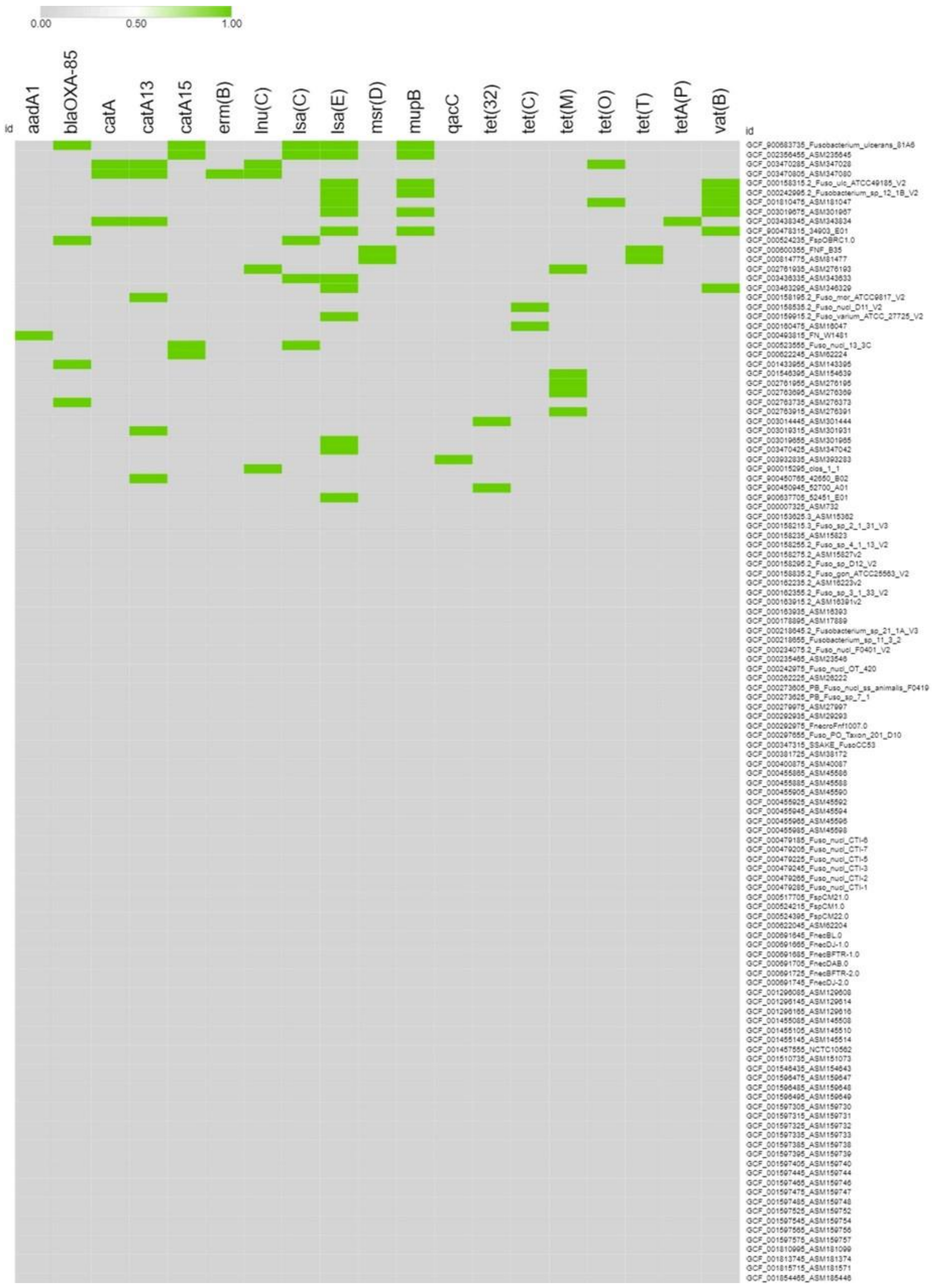
0.00
0.50
1.00
id
aadA1
blaOXA-85
catA
catA13
catA15
erm(B)
lnu(C)
lsa(C)
lsa(E)
msr(D)
mupB
qacC
tet(32)
tet(C)
tet(M)
tet(O)
tet(T)
tetA(P)
vat(B)
id
GCF_900683735_Fusobacterium_ulcerans_81A6
GCF_002356455_ASM235645
GCF_003470285_ASM347028
GCF_003470805_ASM347080
GCF_000158315.2_Fuso_ulc_ATCC49185_V2
GCF_000242995.2_Fusobacterium_sp_12_1B_V2
GCF_001810475_ASM181047
GCF_003019675_ASM301967
GCF_003438345_ASM343834
GCF_900478315_34903_E01
GCF_000524235_FspOBRC1.0
GCF_000600355_FNF_B35
GCF_000814775_ASM81477
GCF_002761935_ASM276193
GCF_003436335_ASM343633
GCF_003463295_ASM346329
GCF_000158195.2_Fuso_mor_ATCC9817_V2
GCF_000158535.2_Fuso_nucl_D11_V2
GCF_000159915.2_Fuso_varium_ATCC_27725_V2
GCF_000160475_ASM16047
GCF_000493815_FN_W1481
GCF_000523555_Fuso_nucl_13_3C
GCF_000622245_ASM62224
GCF_001433955_ASM143395
GCF_001546395_ASM154639
GCF_002761955_ASM276195
GCF_002763695_ASM276369
GCF_002763735_ASM276373
GCF_002763915_ASM276391
GCF_003014445_ASM301444
GCF_003019315_ASM301931
GCF_003019655_ASM301965
GCF_003470425_ASM347042
GCF_003932835_ASM393283
GCF_900015295_clos_1_1
GCF_900450765_42650_B02
GCF_900450945_52700_A01
GCF_900637705_52451_E01
GCF_000007325_ASM732
GCF_000153625.3_ASM15362
GCF_000158215.3_Fuso_sp_2_1_31_V3
GCF_000158235_ASM15823
GCF_000158255.2_Fuso_sp_4_1_13_V2
GCF_000158275.2_ASM15827v2
GCF_000158295.2_Fuso_sp_D12_V2
GCF_000158835.2_Fuso_gon_ATCC25563_V2
GCF_000162235.2_ASM16223v2
GCF_000162355.2_Fuso_sp_3_1_33_V2
GCF_000163915.2_ASM16391v2
GCF_000163935_ASM16393
GCF_000178895_ASM17889
GCF_000218645.2_Fusobacterium_sp_21_1A_V3
GCF_000218655_Fusobacterium_sp_11_3_2
GCF_000234075.2_Fuso_nucl_F0401_V2
GCF_000235465_ASM23546
GCF_000242975_Fuso_nucl_OT_420
GCF_000262225_ASM26222
GCF_000273605_PB_Fuso_nucl_ss_animalis_F0419
GCF_000273625_PB_Fuso_sp_7_1
GCF_000279975_ASM27997
GCF_000292935_ASM29293
GCF_000292975_FnecroFnf1007.0
GCF_000297655_Fuso_PO_Taxon_201_D10
GCF_000347315_SSAKE_FusoCC53
GCF_000381725_ASM38172
GCF_000400875_ASM40087
GCF_000455865_ASM45586
GCF_000455885_ASM45588
GCF_000455905_ASM45590
GCF_000455925_ASM45592
GCF_000455945_ASM45594
GCF_000455965_ASM45596
GCF_000455985_ASM45598
GCF_000479185_Fuso_nucl_CTI-6
GCF_000479205_Fuso_nucl_CTI-7
GCF_000479225_Fuso_nucl_CTI-5
GCF_000479245_Fuso_nucl_CTI-3
GCF_000479265_Fuso_nucl_CTI-2
GCF_000479285_Fuso_nucl_CTI-1
GCF_000517705_FspCM21.0
GCF_000524215_FspCM1.0
GCF_000524395_FspCM22.0
GCF_000622045_ASM62204
GCF_000691645_FnecBL.0
GCF_000691665_FnecDJ-1.0
GCF_000691685_FnecBFTR-1.0
GCF_000691705_FnecDAB.0
GCF_000691725_FnecBFTR-2.0
GCF_000691745_FnecDJ-2.0
GCF_001296085_ASM129608
GCF_001296145_ASM129614
GCF_001296165_ASM129616
GCF_001455085_ASM145508
GCF_001455105_ASM145510
GCF_001455145_ASM145514
GCF_001457555_NCTC10562
GCF_001510735_ASM151073
GCF_001546435_ASM154643
GCF_001596475_ASM159647
GCF_001596485_ASM159648
GCF_001596495_ASM159649
GCF_001597305_ASM159730
GCF_001597315_ASM159731
GCF_001597325_ASM159732
GCF_001597335_ASM159733
GCF_001597385_ASM159738
GCF_001597395_ASM159739
GCF_001597405_ASM159740
GCF_001597445_ASM159744
GCF_001597465_ASM159746
GCF_001597475_ASM159747
GCF_001597485_ASM159748
GCF_001597525_ASM159752
GCF_001597545_ASM159754
GCF_001597565_ASM159756
GCF_001597575_ASM159757
GCF_001810995_ASM181099
GCF_001813745_ASM181374
GCF_001815715_ASM181571
GCF_001854465_ASM185446

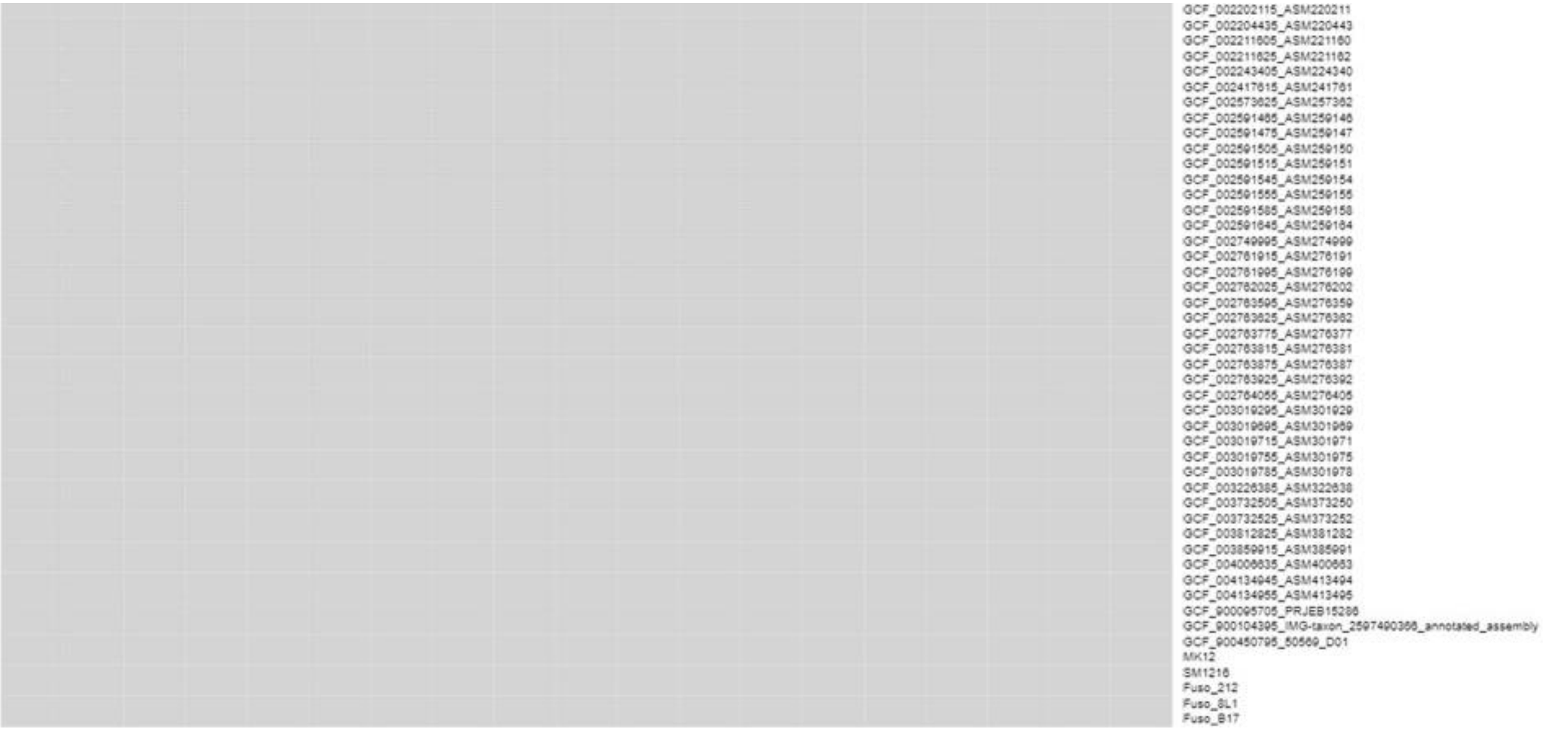
GCF_002202115_ASM220211
GCF_002204435_ASM220443
GCF_002211605_ASM221160
GCF_002211625_ASM221162
GCF_002243405_ASM224340
GCF_002417615_ASM241761
GCF_002573625_ASM257362
GCF_002591465_ASM259146
GCF_002591475_ASM259147
GCF_002591505_ASM259150
GCF_002591515_ASM259151
GCF_002591545_ASM259154
GCF_002591555_ASM259155
GCF_002591585_ASM259158
GCF_002591645_ASM259164
GCF_002749995_ASM274999
GCF_002761915_ASM276191
GCF_002761995_ASM276199
GCF_002762025_ASM276202
GCF_002763595_ASM276359
GCF_002763625_ASM276362
GCF_002763775_ASM276377
GCF_002763815_ASM276381
GCF_002763875_ASM276387
GCF_002763925_ASM276392
GCF_002764055_ASM276405
GCF_003019295_ASM301929
GCF_003019695_ASM301969
GCF_003019715_ASM301971
GCF_003019755_ASM301975
GCF_003019785_ASM301978
GCF_003226385_ASM322638
GCF_003732505_ASM373250
GCF_003732525_ASM373252
GCF_003812825_ASM381282
GCF_003859915_ASM385991
GCF_004006635_ASM400663
GCF_004134945_ASM413494
GCF_004134955_ASM413495
GCF_900095705_PRJEB15286
GCF_900104395_IMG-taxon_2597490366_annotated_assembly
GCF_900450795_50569_D01
MK12
SM1216
Fuso_212
Fuso_8L1
Fuso_B17
